# Trot Accelerations of Equine Front and Hind Hooves Shod with Polyurethane Composite Shoes and Steel Shoes on Asphalt

**DOI:** 10.3390/ani9121119

**Published:** 2019-12-11

**Authors:** Lauren Veneta Moore, Rebeka Roza Zsoldos, Theresia Franziska Licka

**Affiliations:** 1 University Equine Hospital, Department for Companion Animals and Horses, University of Veterinary Medicine Vienna, A-1210 Vienna, Austria; lauren.moore@outlook.com; 2 University of Natural Resources and Life Sciences, A-1190 Vienna, Austria; r.zsoldos@uq.edu.au; 3 Royal (Dick) school of Veterinary Studies, University of Edinburgh, Edinburgh EH8 9YL, UK

**Keywords:** horse, hoof, horseshoe, polyurethane, steel, trot, asphalt

## Abstract

**Simple Summary:**

In the present study, the acceleration occurring during trot on asphalt with two types of horseshoes were compared in horses commonly used for carriage driving in the city of Vienna. Both types of shoes were nailed onto the hooves; one shoe was a traditional steel shoe, while the other one was a steel shoe whose ground surface was covered with soft polyurethane (PU). Four horses were used to measure hoof accelerations during trotting in hand on an asphalt track, similar to a city street. With the polyurethane-covered shoes, hooves experienced less abrupt deceleration during landing; moreover, they also experienced more acceleration after push off from the ground. Front and hind hooves showed similar accelerations when shod with the polyurethane-covered shoe, while front hooves were undergoing harder deceleration than hind hooves when shod with the traditional steel shoe. Finally, with the softer shoes horses trotted faster and with longer strides than with the steel shoes. This indicates that PU shoes may aid in reducing the overload present in the front limbs of horses.

**Abstract:**

The present study investigated accelerations of the front and hind hooves of horses comparing two different shoe types. A standard steel shoe, with studs, pins, and in some instances with toe grabs, was compared to a steel shoe covered on the bottom with a layer of polyurethane. Four horses were used; they trotted in hand on an asphalt track at their self-selected speed. The results showed significantly reduced decelerations during the stance phase with the polyurethane-covered shoes (10th percentile median steel −2.77 g, polyurethane −2.46 g; *p =* 0.06) and significantly increased decelerations in front hooves compared to hind hooves with steel shoes (70th percentile median −1.04 g front hooves, 0.12 g hind hooves, *p* = 0.04). Horses trotted faster using longer strides with the polyurethane-covered shoes compared to the steel shoes. The results show that effects of shoe types should be investigated simultaneously in front and hind hooves, and that PU shoes may aid in reducing the overload present in the front limbs of horses.

## 1. Introduction

Horses play an important role for humans [1]. Different from other species used for, e.g., meat, milk and eggs or wool, the domestication of horses was mainly based on the need for work in the field, as well as transport of goods and humans. In order to meet these needs, horses were selected early on for their locomotion. This is still the most important selection criterion in horse breeding today, as horses are kept for work as well as for athletic purposes [1]. Evaluation and measurement of equine locomotion has a long history. Eadweard Muybridge carried out one of the very first scientific investigations taking pictures of a cantering horse, and he showed that there is a phase during canter where all four legs are off the ground [2]. In livestock farming, accelerometry facilitates lameness detection and provides information about movement in small ruminants and dairy cows [3,4]. For accelerometry of horses, accelerometer devices have been placed on the sternum [5,6], on the dorsal hoofwall [7], or even sandwiched between hoof and horseshoe [8]. A number of studies have reported on the accelerometry of either one front hoof or both front hooves [5,9,10,11], with only one study reporting accelerations of all four hooves [12]. Anatomical markers and video-based motion analysis systems have been used in combination with accelerometry [5,13]. Comparing these methods for the investigation of hoof slippage on soft ground, accelerometry has been shown to be superior to kinematic techniques [14]. In recent years, accelerometers have been commonly combined with inertial sensors, which have become more and more popular for gait and locomotion analysis. Nonetheless, because of the ease of use of the small, light, and wireless accelerometers, and the distinct motion patterns measured, accelerometry is still very popular. The equine hoof is an ideal location to investigate acceleration changes associated with shoeing [11] as the hoof of the horse is subject to marked accelerations and decelerations during locomotion. These depend on the velocity of the horse, the velocity of the hoof during the swing phase, the mass of the hoof (including the shoe if shod), and on the quality of the ground surface where the locomotion takes place. Therefore the accelerations associated with different types of shoes should be compared in the setting that they are intended for.

Using the horse for long distances and every day makes it necessary to prevent excessive hoof wear. In the 15th century B.C., the first form of hoof protection was to cover the sole with variable materials, while in the 1st century B.C., the sole was covered with a metal plate [15]. When steel shoes were adopted, additional features of horse shoes were seen, such as studs and toe grabs [16]. Studs are widely used in horses working on soft surfaces, while their use on hard surfaces changes the angle of the hoof to the ground during weight bearing with increased pressure on the heels and some instability at push off [17]. A single stud was found to markedly reduce slippage on soft ground; however, compared to a medial and a lateral stud, it might create foot rotation [18]. While toe grabs are used to provide grip to the horse on slippery surfaces, they are also a potential risk for injuries caused by stumbling if the toe grab is caught in the ground, or by direct contact between a limb and the grab [19]. Slipping on hard surfaces, such as concrete, is very much dependent on the material properties of the shoe itself, as was shown when comparing shoes with flat ground surfaces made of rubber, steel, or plastic [9].

Besides the forces exerted between the foot and the ground, grip has been described as a key limit to high speed performance in a recent review [20]. Up to the present day, many horses used for carriage driving are shod with horseshoes with grabs in the toe area for better grip, which is permissible only for horses not in ridden use, as it increases the risk of falling [17]. Different athletic uses of horses require specific horseshoes, and different materials for such shoes are in use. Racehorses wear light aluminum horseshoes, while horses kept in groups are often shod with horseshoes made from or covered by softer synthetic materials in order to reduce the risk of kick injuries [21].

In the city of Vienna, on cobbled streets, asphalt, concrete, and a variety of other surfaces, two-horse-drawn carriages of the Landauer type with a carriage mass of about 600–800 kg are common. The majority of horses used for this are shod with steel shoes with a variety of antislip features, such as toe grabs, studs, and pins. However, recently, horses wearing a variety of synthetic shoes are doing the same work, as materials beyond steel have the potential to provide grip [9]. Because of this, the City of Vienna initiated a study focusing on the equine health aspects comparing a routinely used shoe type with a less frequently used, more modern type of shoe. Additionally this study investigated the damage these shoe types create on asphalt surfaces as expensive repairs of city streets are regularly attributed to the carriage horses. The opportunity arose to measure the accelerations of the hooves shod with the two types of horse shoes within the larger study set up. While many synthetic shoes are glued to the hoof, and this may alter the heel movement [22], a shoe, which could be secured on the hoof using nails similar to the standard steel shoe, was chosen. Therefore, the standard carriage shoes traditionally used were compared to a modern steel shoe also nailed to the hoof but whose ground surface is covered with polyurethane.

The aim of the present study was to investigate the differences in hoof accelerations of all four hooves shod with the two types of shoes during trot on an asphalt surface. The first hypothesis investigated was that the polyurethane-covered shoes would significantly change the deceleration during landing as well as the acceleration after push-off. The second hypothesis was that these significant differences would be found similarly in front limbs and in hindlimbs.

## 2. Materials and Methods

This study was discussed and approved by the institutional ethics and animal welfare committee in accordance with GSP guidelines and national legislation (ETK-09/12/2016).

### 2.1. Horses

A convenience sample of four horses (two Standardbred Trotters and two Hungarian Warmbloods; aged 12, 12, 15, 16 years, three geldings, one mare; body mass 465 kg, 483 kg, 500 kg, 506 kg) was used for the present study in a crossover design with both types of shoes with a one week washout period in between. Prior to and after the study, all of the horses were in regular use for carriage driving in the city of Vienna, usually every second day with rest days in between, with 2–6 h of walk and trot per day. A typical Viennese carriage has four wheels and an approximate weight of 600–800 kg. All horses in the present study were deemed adequately sound for their (regular) use as evaluated by the drivers and by the official veterinarians of the city of Vienna, who control the health of these horses. At the start of the study, each horse underwent a gait evaluation as well as a clinical examination of the locomotory system. An experienced equine orthopedic surgeon (T.F.L.) evaluated the gait of the horses. Evaluation took place before the measurements on data collection day (DCD) 1 and DCD 2, as horses graded to show a lameness of more than 1/5 would have been excluded from the study. The day after DCD 1 and DCD 2, horses were again evaluated, as seen in Table 1.

### 2.2. Horseshoes

**Figure 1:**
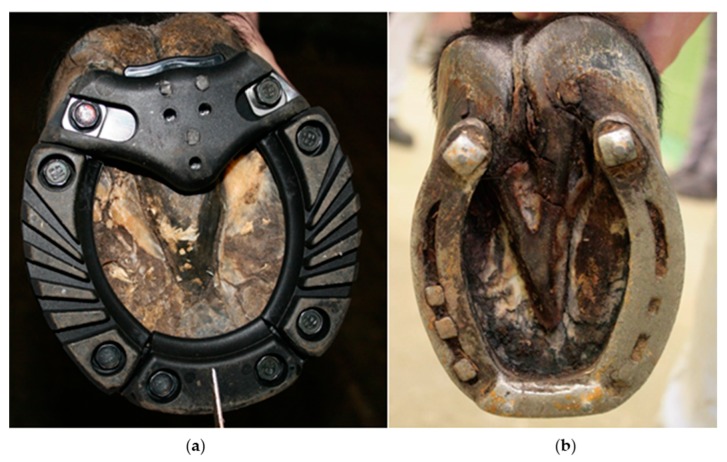
Horseshoe types used in the present study. (**a**) A steel shoe covered with screwed-on polyurethane on the ground surface, a front shoe is shown in the unused condition; (**b**) A traditional carriage-driving steel shoe with toe grab, two studs, and four pins (two of them in the toe grab and one in each stud) of a hind shoe in used condition.

This study compared two different types of horseshoes, as seen in Figure 1; they were studied on two data collection days (DCD) one week apart.

Polyurethane shoes (PU) are composed of a steel shoe and polyurethane segments (Orthoprene^®^, Shore A 90) which are screwed onto the ground surface of the steel shoe, once this is nailed onto the hoof. The frog segment has to be adapted to the horses hoof. The second investigated horseshoe was a traditional carriage steel shoe (ST) with studs and pins in the branches integrated to the shoe as well as grabs and pins in the toe area of the shoe. The masses of the two types of horseshoes are shown in Table 2.

Two horses had ST with grabs and pins (horses I, III) and two had only pins and no grabs in the toe area (horses II, IV). All ST shoes had studs and pins at the end of the shoe branches. The pins in the studs had a diameter of 5 mm and a height of 3 mm, the studs themselves were 10 mm high and the toe grabs with integrated pins were 6 mm high.

### 2.3. Shoeing

All four horses were shod with PU the day before DCD 1 after trimming and balancing the feet routinely. Based on farrier availability, two horses were shod by their own farrier, while the other two horses were shod by the farrier from the University of Veterinary Medicine Vienna (Vetmeduni Vienna). The day before DCD 2, all horses were shod with ST by their own farriers, but the hooves were not again trimmed and balanced.

### 2.4. Accelerometry

Two different accelerometer systems were used in the present study. The first accelerometer system was the Xsens Mtw (Xsens Inc., Enschede, The Netherlands) with accelerometer mass of 27 g, dimensions of 34.5 × 57.8 × 14.5 mm, and a sampling rate of 50 Hz for acceleration data. The measurement range of this system was ± 16 g. During the measurement of the first horse (horse IV) with this system, difficulties with data transmission to the base station were encountered, possibly because the ambient temperature was below the recommended range. Therefore from the second horse on the first measurement day onwards, the system was changed to Delsys^®^ Trigno™ Sensors (Delsys Inc., Natick, MA, USA) with accelerometer mass of 14 g, size of 37 × 26 × 15 mm, and a sampling rate of 148 Hz for acceleration data. The detectable range for each axis was 4 g (−2 g to +2 g), resulting in a total range of the sum of the measured *x*-*, y*-*,* and *z*-accelerations of 12 g (−6 g to +6 g). The values obtained using the Xsens Mtw were converted from m/s^2^ to g, and a cut off similar to the measurement cut off of the Delsys accelerometers was used for further data processing. A repeat measurement of horse IV with the Delsys accelerometers was not possible, as these measurements were embedded into a day of trotting horses 2100 lengths in hand on the same examination track to document the wear of the asphalt of the examination track.

**Figure 2:**
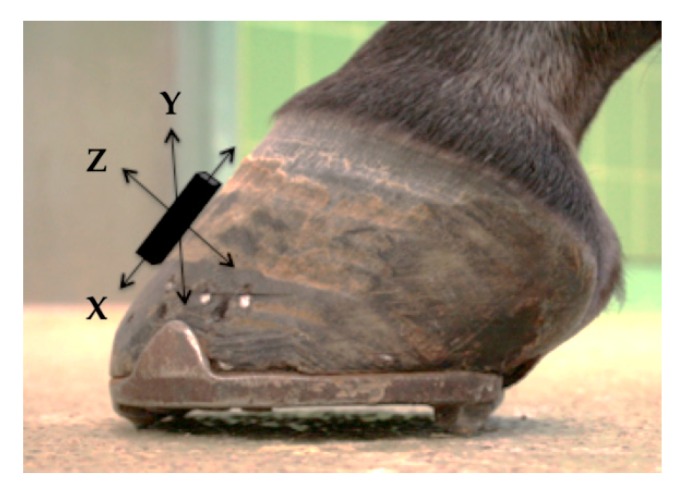
Schematic depiction of the accelerometer location on the dorsal hoof wall with the axes measured: *x*-axis proximodistal, *y*-axis mediolateral, and *z*-axis dorsopalmar/dorsoplantar.

On each hoof, accelerometers were mounted on the dorsal hoof wall, as seen in Figure 2, with textile adhesive tape. Both accelerometer types are triaxial, and they were mounted so that the axes of the accelerometer were consistent; *x*-axis measured accelerations in proximodistal direction, *y*-axis accelerations in mediolateral direction, and *z*-axis measured accelerations in the dorsopalmar/-plantar direction and transmitted data to the base station. Beyond the built-in processing of the accelerometer base stations, no further filtering was used.

### 2.5. Data Collection

Data collection took place on two data collection days (DCD 1 December 13th; DCD 2 December 20th, 2016) one week apart at the Vetmeduni Vienna. During data collection, ambient temperature ranged between +1 °C and −4 °C. Prior to data collection, the asphalt of the examination track had been replaced with a 15 cm thick layer of asphalt regularly used in the city of Vienna (technical specification in line with Municipal Department 28—Road Management and Construction (MA 28), Vienna, Austria), for the exclusive use in the present study. The track had a length of 40 m; 32 m were used for trotting, and at the beginning and at the end of the track 4 m were used for turning the horses. If a horse defecated during data collection, fecal matter was immediately removed, so that the horses were continuously trotting directly on the uncovered asphalt.

For data collection, horses were trotted in hand by volunteer students at the Vetmeduni Vienna who had successfully completed initial training in horse handling. Horses were led from the left side with a lead rope attached to the head collar, and they always turned to the right. Each horse was allowed to trot at its preferred velocity, with the handler adapting to this velocity. Horses were measured in following order: DCD 1 Horse IV, I, II, III, and on Day 2 Horse IV, II, I, III. As the trotting order was not deemed to be influential, this was not controlled, but horses were presented in the order in which they were stabled. Measurements were taken over 5 min, with approximately 20 lengths of trot and the turns between these lengths recorded.

### 2.6. Data Processing

**Figure 3:**
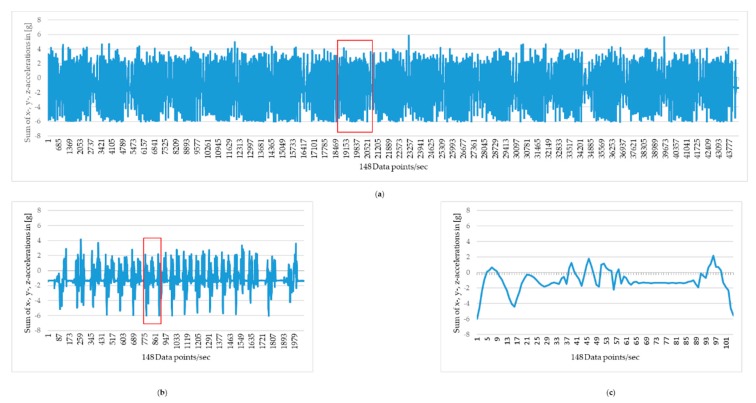
Sum of the measured *x*-, *y*-, and *z*-accelerations of the left front hoof of horse I shod with a polyurethane (PU)-covered shoe on the first measurement day. (**a**) The graph shows 20 lengths of trot on a 40 m asphalt track; each length of trot, approximately 32 m, is identified by the two walk and turn events, approximately 4 m, preceding and following it; (**b**) This graph shows the single length of trot, which is located within the red box in the top graph; (**c**) This graph shows the single motion cycle of trot between two local acceleration minima, which is located within the red box in the bottom left graph. Red boxes are slightly larger than the length or motion cycle respectively, to reduce overlap with the relevant aspects of the graph.

The use of percentiles for the presentation of the accelerations in the present study was based on the calibration ranges of the accelerometry system used, as true maxima and minima of hoof accelerations would have been beyond this range; therefore, percentiles (minimum 10th and maximum 90th) were chosen to illustrate differences in accelerations without relying on minima and maxima. The measured *x*-, *y*-, and *z*-accelerations of the three axes were used to calculate the sum of the *x*-, *y*-, and *z*-accelerations for each accelerometer. Data plots were used to split the measurement of every hoof into trotting lengths, using the obviously lower accelerations and the slower motion cycles during walk and turning to identify the lengths, as seen in Figure 3. One length consisted of up to 20 motion cycles, including one to three motion cycles in walk before/after trot including the turn; and this was used for separating the trotting lengths. For each horse, a minimum of 11 lengths with all trotting motion cycles (118 to 154 motion cycles per horse limb) were further processed.

Local minima were identified to split the trace of each length into motion cycles of trot. Each motion cycle was defined to start at and end one data point before a local minimum at the beginning of the stance phase, representing maximum deceleration on hoof landing. Data plots were used to select at least 10 motion cycles of trot per length for statistical analysis. As the measurement range of the accelerometers used is markedly smaller than the expected real accelerations of the hooves, acceleration data were analyzed for their percentiles rather than for minima and maxima. Of the sum of the x, y, and z accelerations, 10–90th percentiles were calculated for each motion cycle; and of all motion cycles for each length the median of each of these percentile values was taken forward for statistical analysis.

The velocity for each length considered was calculated as m/s using the time needed for the 32 m of trotting length.

### 2.7. Statistical Analysis

Statistical analysis was performed in SPSS (Version 24, IBM, Armonk, NY, USA). As acceleration data were not normally distributed, based on the Shapiro–Wilk test, the Wilcoxon singed rank test for nonparametric data was used for comparisons. Significance was set at *p* ≤ 0.05. In order to investigate whether the relationship between velocity, which was normally distributed, and acceleration was similar with the two types of shoes, Pearson correlation coefficients were calculated. Furthermore, the relationship between acceleration percentiles and the ordinal numbers of the eleven lengths of trot used was described calculating Pearson correlation coefficients between these parameters. This was done to investigate potential habituation to the trotting situation and/or the shoes over the measurement period. Correlations were reported if R^2^ ≥ 0.4. The masses of the shoes used were compared in a Mann–Whitney U test. Testing for a potential influence of trotting order, the accelerometry results were ranked and compared to the trotting order. Similarly, the accelerometry results of the ST shoes with and without toe grabs were ranked to identify a possible influence of the toe grab.

## 3. Results

The shoes used for the present study did not have significantly different masses. Ranking the accelerometry results of the horses and comparing these ranks to the order in which they were measured showed that the order of measurement had no apparent influence on the accelerometry results.

In Table 3, the number of motion cycles used per horse and per hoof are listed. In three horses, 11 consecutive lengths, and in one horse, due to the loss of transmission of one accelerometer during the measurement, only nine consecutive lengths of trot were available, a total of 42 lengths were available for the ST condition. Moreover, in the ST condition, the accelerometer on the left hind hoof of Horse 3 was dysfunctional throughout the measurement on DCD 2; therefore, no data were available for this hoof in this condition. Because lengths with irregular or stumbling strides were unsuitable to determine true trotting velocity, 163 lengths in the PU condition and 159 lengths in the ST condition were used for these calculations. These stumbling or irregular strides lead to the removal of seven motion cycles in the ST condition and 26 motion cycles in the PU condition from further analysis.

### 3.1. Velocities

Horses trotted significantly (*p* = 0.001) faster with PU shoes (median 4.15 m/s, range 3.36–4.55 m/s) than with ST shoes (median 3.67 m/s, range 3.32–4.47 m/s). For the 32 m lengths used for the determination of trotting velocities, the horses used significantly (*p* < 0.001) fewer strides with the PU (median 12.25, range 11–14 strides) than with the ST shoes (median 13 strides, range 11–15 strides).

### 3.2. Accelerations

Ranking the accelerometry results of the ST condition with and without toe grabs, these did not form two distinct groups. Over the front and hind hooves, significant differences were found in percentile acceleration values; hooves shod with PU showed less marked decelerations (percentile 10–70th) and more marked accelerations (percentile 80–90th) than with hooves shod with ST, as seen in Table 4.

Considering front and hind hooves separately, the overall differences can be attributed to differences in the 10–90th percentile accelerations values of the front hooves, as there were no significant differences in hind hoof accelerations between the two shoe types. Between front and hind hooves shod with PU no significant differences were found in the 10–90th percentile accelerations values, as seen in Table 5.

Over all the lengths considered for each horse, there were few significant correlations between the ordinal numbers of these lengths and the percentiles of acceleration values, as seen in Supplementary Table S1. Over all the lengths considered for each horse between the mean velocities and the ordinal numbers of these lengths, correlations were found for Horse III in the left hind hoof shod with PU (−0.15, R^2^ = 0.6). More correlations between the ordinal number of these lengths and the mean velocities were found in ST condition for Horse I in the right hind hoof (0.9, R^2^ = 0.5) and Horse IV in the left and right front hooves (−0.10, R^2^ = 0.5; −0.15, R^2^ = 0.5; median = 3.67 m/s; and right hind hoof (−0.15, R^2^ = 0.5). Significant correlations between velocities and acceleration values were commonly present for both types of horseshoes, as seen in Supplementary Table S2.

## 4. Discussions

This study is the first to present an analysis of the effects of two different types of shoes on accelerations during extended duration of trot in hand, and resulted in a large number of motion cycles available for this investigation. While previous studies have investigated the effects of different shoes on front and hindlimbs [23], it is actually much more common to use the same type of shoes in front and hind feet, as was the case in the present study. The large number of motion cycles measured allowed detection of some adaptation effects during the measurements. The hypothesis investigated, i.e., that there would be significant differences in trotting accelerations between the two shoe types, could be wholly confirmed. The result that a polyurethane ground surface of a horseshoe is associated with less marked decelerations and more marked accelerations during trot compared to steel was expected based on the elastic and soft quality of the material. This effect of polyurethane has been shown in a study in front feet [24].

Considering the faster trotting velocity with the PU shoe, the differences in reduced deceleration become even more relevant, while the differences in acceleration may (partly) be the consequence of this. This faster velocity, combined with the short habituation time of the horses to the PU shoe (one day only), may have contributed to the slightly higher number of motion cycles that were irregular (and thus excluded from statistical analysis). The horses had been wearing ST shoes regularly for years, and with these, there was a much smaller number of irregular motion cycles during the slightly slower trot.

The absence of significant differences between shoe types in the hind hooves is of interest as it again indicates that the use of the hind hooves is very different regarding the acceleration effects of horse shoes. Moreover, the even distribution between front and hind hoof accelerations in horses shod with PU shoes creates further questions regarding the effect of shoeing on the use of the whole body during locomotion. It is interesting to note that in earlier studies, differences in front and hind feet forces were found, but these differences could not be shown using simultaneous accelerometry [12,25].

Clearly, the present study has a number of relevant limitations: The horses were measured trotting in hand and during continuous trot only; during carriage driving, different accelerations have to be expected; this is also true for the start of locomotion, breaking, and turning. Only two types of horse shoes were compared. The PU shoes required some trimming of the frog, and this was still notable when the ST shoes were placed one week later, with an unknown effect. Furthermore, the ST shoes were used in two slight variations (with and without toe grabs, with the ground contact in both effected by the pins in the toe region of the shoe or in the toe grab itself). Effects of shortening breakover have been documented [26], and it is conceivable that pins in the toe region of the shoe would create the opposite effect of delaying breakover. However, no assumptions of the difference of the effects of pins in the toe region of the shoe and of pins in the toe grab itself can be made. The shoes were all placed by experienced and well-trained farriers, and the comparisons were made without additional trimming and balancing of the hooves, as there was only one week between the shoeings. However, no attempt was made to document the foot shapes of the horses, even though this, similar to the balance achieved by trimming, has a relevant effect on the accelerations measured [27].

Ranking of the accelerometry results of the ST shoes did not indicate an effect of the presence of a toe grab, as these shoes did not rank together, but this may be due to too small numbers investigated. The reason for using the ST shoes in their two variations was the traditional use of both of these variations in the carriage horses in the city of Vienna, and secondly, the need to investigate both shoes as part of a previously described larger study. Choosing a shoe for comparison with the ST shoe was done trying to limit the differences to the ST shoe beyond the more elastic and soft ground surface; therefore, a second shoe with a steel base nailed onto the hoof was chosen. A more extreme choice of comparison, e.g., with a glue-on, fully synthetic shoe can be expected to produce much more marked differences, but such findings could then not be attributed to a distinct differing feature, i.e., the quality of the ground surface of the shoe, as was the case in the current comparison. The number of horses used for the present study was low, and the investigation of a larger group of horses is preferred for biomechanical studies.

In the present study, the asphalt surface used was the same as that covering large parts of the streets where carriages are driven through the city of Vienna. The results will therefore not be comparable to results obtained on softer surfaces, or on a treadmill, which has been used in many investigations into equine locomotion. Clearly, the advantages of treadmill use are numerous—a set speed of locomotion, and the possibility to combine several measurement techniques in a small measurement space. For the present study, a more realistic scenario was used by having the horses choose their own trotting speed. This allowed us to document that, while the trotting speed was different, the number of motion cycles used for each length was not significantly different; therefore, horses mainly made slower strides when shod with ST shoes than when shod with PU shoes. It would be of great interest to associate this finding with the level of comfort that the horses experienced when trotting with either of the two shoe types; unfortunately, the present study did not investigate this complex, but relevant question. One indication may be the level of soundness on the day after the measurements, which showed two more horses to be sound in the forelimbs after the use of the PU shoes, while no such difference was found with the steel shoes; however, more horses and a more sophisticated and objective assessment of lameness should be used.

## 5. Conclusions

The present study shows the need to investigate effects of horseshoes in all four limbs, as during trot in hand on asphalt, the PU shoes led to a more even distribution of accelerations between fore and hindlimbs than the ST shoes. This indicates that PU shoes may aid in reducing the overload present in the front limbs of horses. Future studies are needed to investigate this effect during riding and on softer surfaces.

## Figures and Tables

**Table 1:** Results of the gait examination of the four horses. Lameness grades presented are the mean of two lengths of trot assessed; grade 0—lameness not perceptible, grade 1—lameness difficult to observe and not consistently apparent I = Horse 1, II = Horse 2, III = Horse 3, IV = Horse. DCD 1 = Data collection day 1, DCD 2 = Data collection day 2. FR = front right, FL = front left, HR = hind right, HL = hind left.

Horse	DCD 1		Day after DCD 1	
I	FL 0.5/5	HR 1/5	-	-
II	FL 0.5/5	HR 1/5	-	HR 1/5
III	FL 1/5	HR and HL 0/5	FL 0.5/5	HR 1/5
IV	-	-	-	HR 0.5/5
	**DCD 2**		**Day after DCD 2**	
I	FL 0.5/5	HR 1/5	FL 1/5	HL 0.5/5
II	FR 1/5	HR 1/5	FR 1/5	HR 1/5
III	FL 1/5	HR 0.5/5	FL 0.5/5	HR 1/5
IV	FL 0.5/5	HR 1/5	FL 0.5/5	HR 1/5

**Table 2:** Masses in grams of the investigated horseshoes. Shoes were weighed before use and without the nails. Nails (number and type) were similar for both shoe types. Masses for polyurethane shoes (PU) included the basic steel shoe and all of the original polyurethane segments. The frog segment has to be slightly trimmed to fit the hoof during mounting of the polyurethane segments. Masses for the steel shoes (ST) included studs, grabs, and pins.

Shoes	PU		ST	
	Size 3	Size 4	Size 3	Size 4
Front	481 g	508 g	500 g	522 g
Hind	485 g	498 g	505 g	531 g

**Table 3:** Numbers of motion cycles available for analysis in the two shoeing conditions (steel shoe ST, shoe with polyurethane PU) for each of the hooves and all horses. Please be aware that the left hind hoof in the ST shoeing situation of horse 3 is missing. FR = front right, FL = front left, HR = hind right, HL = hind left.

Horse	Shoes	Total	FL	FR	HL	HR
**I**	**PU**	515	135	137	125	118
	**ST**	593	148	153	143	149
**II**	**PU**	520	141	134	121	124
	**ST**	554	154	143	122	135
**III**	**PU**	533	131	139	128	135
	**ST**	428	143	152	0	133
**IV**	**PU**	531	139	135	133	124
	**ST**	529	139	139	130	121

**Table 4:** The 10–90th percentiles of the measured sum of the *x*-, *y*-, and *z*-accelerations, mean values (g) of the median values of 11 lengths with a minimum of 10 motion cycles of trot considered for each length. Please be aware that the left hind hoof in the ST shoeing situation of horse 3 is missing. Values presented are of all hooves of three horses, and of three hooves of one horse (total 15 hooves). Data obtained with a steel shoe with the ground surface covered with a polyurethane layer (PU) and with a traditional steel shoe with pins and studs (ST) are listed. Significance of the difference between horseshoe types is presented (*p*-values). Min = Minimum, Max = Maximum.

Percentile	PU			ST			PU vs. ST
	Median	Min	Max	Median	Min	Max	*p*-value
10th	−2.46	−3.24	−0.78	−2.77	−4.51	−2.09	0.06
20th	−1.72	−2.60	0.31	−1.98	−3.18	−1.58	0.04
30th	−1.41	−1.95	1.01	−1.67	−2.20	−1.33	0.03
40th	−1.38	−1.69	1.36	−1.61	−1.81	−1.11	0.02
50th	−1.24	−1.63	1.44	−1.46	−1.72	−0.33	0.03
60th	−0.73	−1.55	1.49	−1.14	−1.65	0.28	0.04
70th	−0.02	−1.15	1.72	−0.43	−1.31	0.86	0.09
80th	0.68	−0.29	2.01	0.24	−1.41	1.10	0.02
90th	1.86	0.80	2.82	1.32	0.56	2.20	0.04

**Table 5:** **The** 10–90th percentiles of the measured sum of the *x*-, *y*-, and *z*-accelerations, mean values (g) of the median values of 11 lengths with a minimum of 10 motion cycles of trot considered for each length. Please be aware that the left hind hoof in the ST shoeing situation of horse 3 is missing. Values presented are of all hooves of three horses, and of three hooves of one horse (total 15 hooves). Data obtained with a steel shoe with the ground surface covered with a polyurethane layer (PU) and with a traditional steel shoe with pins and studs (ST) is listed. Significance of the difference between front and hind hooves with PU and front and hind hooves with ST is presented as well as differences between PU and ST in front or hind hooves (*p*-Values). F = Front hooves, H = Hind hooves.

Percentile	PU			PU				ST			ST				PU vs. ST	PU vs. ST
	Front			Hind			F vs. H	Front			Hind			F vs. H	Front	Hind
	Median	Min	Max	Median	Min	Max	*p*-Values	Median	Min	Max	Median	Min	Max	*p*-Values	*p*-Values	*p*-Values
10th	−2.33	−3.21	−0.78	−2.81	−3.24	−1.99	0.21	−2.89	−3.54	−2.38	−2.77	−4.51	−2.09	1.00	0.01	0.87
20th	−1.65	−2.60	0.31	−1.81	−2.46	−1.32	0.26	−2.15	−2.71	−1.67	−1.83	−3.18	−1.58	0.74	0.02	0.46
30th	−1.41	−1.94	1.01	−1.39	−1.95	−0.41	0.33	−1.76	−2.20	−1.40	−1.64	−1.83	−1.33	0.50	0.02	0.31
40th	−1.38	−1.65	1.36	−1.37	−1.69	0.45	0.40	−1.58	−1.81	−1.32	−1.61	−1.77	−1.11	0.80	0.01	0.31
50th	−1.29	−1.63	1.44	−1.20	−1.62	1.22	0.78	−1.55	−1.72	−1.29	−1.40	−1.71	−0.33	0.40	0.01	0.40
60th	−0.97	−1.55	1.49	−0.45	−1.48	1.41	0.16	−1.44	−1.65	−0.95	−0.79	−1.55	0.28	0.09	0.03	0.35
70th	−0.41	−1.15	1.72	0.22	−0.84	1.45	0.12	−1.04	−1.31	−0.36	−0.12	−0.75	0.86	0.04	0.09	0.50
80th	0.38	−0.29	2.01	0.97	0.07	1.81	0.12	−0.31	−0.51	0.48	0.61	−1.41	1.10	0.13	0.05	0.18
90th	1.72	0.80	2.67	1.86	0.99	2.82	0.18	0.87	0.61	1.74	1.92	0.56	2.20	0.06	0.06	0.40

## Supplementary Materials

The following are available online at https://www.mdpi.com/2076-2615/9/12/1119/s1, Table S1: Correlations over the ordinal number of the 11 lengths and the 10th -90th Percentiles of the sum total of the hoof accelerations of all 3 axes. Table S2: Correlation between velocity of the horses in the two different horseshoes 10th -90th Percentiles of the sum total of the hoof accelerations of all 3 axes.
