# Supplementary material for: Trot Accelerations of Equine Front and Hind Hooves Shod with Polyurethane Composite Shoes and Steel Shoes on Asphalt
Source: Animals (Basel). 2019 Dec 11;9(12):1119. doi: 10.3390/ani9121119 (PMC6940983; doi:10.3390/ani9121119)
Supplement: Supplementary file 1 [file animals-09-01119-s001.docx]

**Table S1.** Correlations over the ordinal number of the 11 lengths and the 10th -90th Percentiles of the sum total of the hoof accelerations of all 3 axes, mean values [g] of the median values of 11 lengths with a minimum of 10 motion cycles of trot considered for each length. Values presented are of all hooves of three horses, and of three hooves of one horse (total 15 hooves). Data obtained with a steel shoe with the ground surface covered with a polyurethane layer (P) and with a traditional steel shoe with pins and studs (S) is listed for R^2^ ≥ 0.4. CC= Correlation coefficient. LL = Lower limit of the 95% CI, and UL = upper limit of the 95% confidence interval.

|  |  | | 10th Percentile | | | | | | | | | | | | 20th Percentile | | | | | | | | | | | | | | | | | 30th Percentile | | | | | | | | | | | | | | | | | 40th Percentile | | | | | | | | | | | | | | | | | | 50th Percentile | | | | | | | | | | | |  |  |  |  |  |  |  |  |  |  |  |
| --- | --- | --- | --- | --- | --- | --- | --- | --- | --- | --- | --- | --- | --- | --- | --- | --- | --- | --- | --- | --- | --- | --- | --- | --- | --- | --- | --- | --- | --- | --- | --- | --- | --- | --- | --- | --- | --- | --- | --- | --- | --- | --- | --- | --- | --- | --- | --- | --- | --- | --- | --- | --- | --- | --- | --- | --- | --- | --- | --- | --- | --- | --- | --- | --- | --- | --- | --- | --- | --- | --- | --- | --- | --- | --- | --- | --- | --- | --- | --- | --- | --- | --- | --- | --- | --- | --- | --- | --- | --- |
| LF_S | H | | R^2^ | | | CC | | | LL | | | UL | | | | R^2^ | | | CC | | | | LL | | | | | UL | | | | R^2^ | | | | | CC | | | | LL | | | | UL | | | | R^2^ | | | | | | CC | | | | LL | | | | UL | | | | R^2^ | | | CC | | | LL | | | UL | | |  |  |  |  |  |  |  |  |  |  |  |
|  | III | | 0.63 | | | 9 | | | 4 | | | 14 | | | | 0.59 | | | *12* | | | | 5 | | | | | 20 | | | | 0.59 | | | | | *18* | | | | *7* | | | | *29* | | | | 0.87 | | | | | | 31 | | | | *22* | | | | 40 | | | | 0.61 | | | *20* | | | 8 | | | 32 | | |  |  |  |  |  |  |  |  |  |  |  |
|  | II | |  | | |  | | |  | | |  | | | |  | | |  | | | |  | | | | |  | | | |  | | | | |  | | | |  | | | |  | | | | 0.61 | | | | | | 282 | | | | *112* | | | | 451 | | | |  | | |  | | |  | | |  | | |  |  |  |  |  |  |  |  |  |  |  |
|  |  | | 40th Percentile | | | | | | | | | | | | | 50th Percentile | | | | | | | | | | | | | | | | 60th Percentile | | | | | | | | | | | | | | | | |  |  |  |  |  |  |  |  |  |  |  |  |  |  |  |  |  |  |  |  |  |  |  |  |  |  |  |  |  |  |  |  |  |  |  |  |  |  |  |  |  |
| RF_S | IV | |  | | |  | | |  | | |  | | | |  | | |  | | | |  | | | | |  | | | | 0.46 | | | | | *-208* | | | | *-378* | | | | *38* | | | |  |  |  |  |  |  |  |  |  |  |  |  |  |  |  |  |  |  |  |  |  |  |  |  |  |  |  |  |  |  |  |  |  |  |  |  |  |  |  |  |  |
|  | I | | 0.77 | | | 425 | | | 251 | | | 599 | | | | 0.78 | | | *487* | | | | 292 | | | | | 681 | | | |  | | | | |  | | | |  | | | |  | | | |  |  |  |  |  |  |  |  |  |  |  |  |  |  |  |  |  |  |  |  |  |  |  |  |  |  |  |  |  |  |  |  |  |  |  |  |  |  |  |  |  |
|  |  | | 30th Percentile | | | | | | | | | | | | | 40th Percentile | | | | | | | | | | | | | | | | 50th Percentile | | | | | | | | | | | | | | | | | 60th Percentile | | | | | | | | | | | | | | | | | | 70th Percentile | | | | | | | | | | | | | 80th Percentile | | | | | 90th Percentile | | | | |
| LH_S | IV | | | | 0.53 | | | *14* | | 4 | | | 24 | | | | |  | | | |  | | | | |  | | | |  | | | | |  | | | |  | | | |  | | | |  | | | | | | 0.48 | | | | 297 | | | | *62* | | | | 531 | | |  | | |  | | |  | | |  |  | | |  |  |  | |  |  |  |  |
|  | III | | | |  | | |  | |  | | |  | | | | | 0.66 | | | | *118* | | | | | 55 | | | | 182 | | | | | 0.69 | | | | *27* | | | | *41* | | | | *13* | | | | | | 0.66 | | | | 19 | | | | *30* | | | | 9 | | | 0.65 | | | *16* | | | 24 | | | 7 | 0.60 | | | 19 | 31 | 7 | | 0.40 | -12 | -23 | 1 |
|  | II | | | |  | | |  | |  | | |  | | | | |  | | | |  | | | | |  | | | |  | | | | | 0.94 | | | | *-22* | | | | *-26* | | | | *-18* | | | | | | 0.72 | | | | -13 | | | | *-19* | | | | -7 | | | 0.74 | | | *-15* | | | -22 | | | -8 | 056 | | | -12 | -21 | -4 | |  |  |  |  |
| RH_S |  | | | | 10th Percentile | | | | | | | | | | | | | 20th Percentile | | | | | | | | | | | | | | | | | | 30th Percentile | | | | | | | | | | | | | | | | | | 40th Percentile | | | | | | | | | | | | | | | 70th Percentile | | | | | | | | | | 80th Percentile | | | | | | | 90th Percentile | | | |
|  | IV | | | |  | | |  | |  | | |  | | | | 0.58 | | | | *10* | | | | | 4 | | | | | 16 | | | | | 0.41 | | | | *11* | | | | *1* | | | | *20* | | | | | | 0.53 | | | | 11 | | | | *3* | | | | 19 | | |  | | |  | | |  | | |  |  | | |  |  |  | |  |  |  |  |
|  | III | | | | 0.77 | | | *20* | | 28 | | | 12 | | | |  | | | |  | | | | |  | | | | |  | | | | |  | | | |  | | | |  | | | |  | | | | | |  | | | |  | | | |  | | | |  | | |  | | |  | | |  | | |  |  | | |  |  |  | |  |  |  |  |
|  | II | | | |  | | |  | |  | | |  | | | |  | | | |  | | | | |  | | | | |  | | | | | 0.87 | | | | *271* | | | | *183* | | | | *348* | | | | | |  | | | |  | | | |  | | | |  | | | 0.44 | | | *-18* | | | -32 | | | -3 | 0.74 | | | -18 | -26 | -10 | | 0.83 | -12 | -16 | -8 |
| LF_F |  | | | | 10th Percentile | | | | | | | | | | | | 30th Percentile | | | | | | | | | | | | | | | | | | | 60th Percentile | | | | | | | | | | | | | | | | | | 70th Percentile | | | | | | | | | | | | | | |  |  |  |  |  |  |  |  |  |  |  |  |  |  |  |  |  |  |  |  |  |
|  | IV | | | | 0.47 | | | *10* | | 2 | | | 18 | | | | 0.43 | | | | *73* | | | | | 10 | | | | | 135 | | | | |  | | | |  | | | |  | | | |  | | | | | | 0.58 | | | | -50 | | | | *-82* | | | | -18 | | |  |  |  |  |  |  |  |  |  |  |  |  |  |  |  |  |  |  |  |  |  |
|  | III | | | | 0.51 | | | *-19* | | -32 | | | -5 | | | |  | | | |  | | | | |  | | | | |  | | | | |  | | | |  | | | |  | | | |  | | | | | |  | | | |  | | | |  | | | |  | | |  |  |  |  |  |  |  |  |  |  |  |  |  |  |  |  |  |  |  |  |  |
|  | II | | | |  | | |  | |  | | |  | | | | 0.44 | | | | *77* | | | | | 12 | | | | | 142 | | | | | 0.42 | | | | *81* | | | | *9* | | | | *153* | | | | | | 0.60 | | | | 44 | | | | *17* | | | | 72 | | |  |  |  |  |  |  |  |  |  |  |  |  |  |  |  |  |  |  |  |  |  |
| RF_F |  | | | | 40th Percentile | | | | | | | | | | | | 60th Percentile | | | | | | | | | | | | | | | | | | 80th Percentile | | | | | | | | | | | | | | | | | |  |  |  |  |  |  |  |  |  |  |  |  |  |  |  |  |  |  |  |  |  |  |  |  |  |  |  |  |  |  |  |  |  |  |  |  |  |
|  | | IV | |  | | |  | | | |  | | |  | | |  | | | |  | | | |  | | | | |  | | | | 0.47 | | | | | *-148* | | | | *-265* | | | | *-30* | | | | |  |  |  |  |  |  |  |  |  |  |  |  |  |  |  |  |  |  |  |  |  |  |  |  |  |  |  |  |  |  |  |  |  |  |  |  |  |  |
|  | | II | | 0,62 | | | *340* | | | | 141 | | | 539 | | | 0.62 | | | | *340* | | | | 141 | | | | | 539 | | | | 0.71 | | | | | *-30* | | | | *-45* | | | | *-16* | | | | |  |  |  |  |  |  |  |  |  |  |  |  |  |  |  |  |  |  |  |  |  |  |  |  |  |  |  |  |  |  |  |  |  |  |  |  |  |  |
| LH_F | |  | | 50th Percentile | | | | | | | | | | | | | 70th Percentile | | | | | | | | | | | | | | | | | 80th Percentile | | | | | | | | | | | | | | | | | 90th Percentile | | | | | | | | | | | | | | | | | |  |  |  |  |  |  |  |  |  |  |  |  |  |  |  |  |  |  |  |  |  |
|  | | IV | |  | | |  | | | |  | | |  | | | 042 | | | | *-15* | | | | -29 | | | | | -2 | | | | 0.66 | | | | | *-14* | | | | -21 | | | | -6 | | | | | 0.44 | | | | | *-10* | | | | *-19* | | | | *-1* | | | |  |  |  |  |  |  |  |  |  |  |  |  |  |  |  |  |  |  |  |  |  |
|  | | I | | 0.55 | | | -36 | | | | *-61* | | | -11 | | |  | | | |  | | | |  | | | | |  | | | |  | | | | |  | | | |  | | | |  | | | | |  | | | | |  | | | |  | | | |  | | | |  |  |  |  |  |  |  |  |  |  |  |  |  |  |  |  |  |  |  |  |  |
| RH_F | |  | | 10th Percentile | | | | | | | | | | | | | 60th Percentile | | | | | | | | | | | | | | | | 70th Percentile | | | | | | | | | | | | | | | | | 80th Percentile | | | | | | | | | | | | | | | | | | 90th Percentile | | | | | | | | | | | | | | | | | | | | | |
|  | | IV | | 0.70 | | | *21* | | | | 11 | | | 32 | | | 0.53 | | | *-18* | | | | -30 | | | | | -5 | | | | 0.80 | | | | | *-25* | | | | *-34* | | | | *-15* | | | | 0.61 | | | | | | -20 | | | | *-33* | | | | -8 | | | | 0.50 | | | *-28* | | | -50 | | | -7 | | | |  |  |  |  |  |  |  |  |  |
|  | | II | |  | | |  | | | |  | | |  | | |  | | |  | | | |  | | | | |  | | | |  | | | | |  | | | |  | | | |  | | | | 0.63 | | | | | | 33 | | | | *14* | | | | 52 | | | | 0.47 | | | *13* | | | 3 | | | 23 | | | |  |  |  |  |  |  |  |  |  |

**Table S2.** Correlation between velocity of the horses in the two different horseshoes 10th -90th Percentiles of the sum total of the hoof accelerations of all 3 axes, mean values [g] of the median values of 11 lengths with a minimum of 10 motion cycles of trot considered for each length. Values presented are of all hooves of three horses, and of three hooves of one horse (total 15 hooves). Data obtained with a steel shoe with the ground surface covered with a polyurethane layer (P) and with a traditional steel shoe with pins and studs (S) is listed for R^2^ ≥ 0.4. CC= Correlation coefficient. LL = Lower limit of the 95% CI, and UL = upper limit of the 95% confidence interval.

|  | | | | 70th Percentile | | | | | | | | | | | |  |  |  |  |  |  |  |  |  |  |  |  |  |  |  |  |  |  |  |  |  |  |  |  |  |  |  |  |  |  |  |  |  |  |  |  |  |  |  |  |  |  |  |  |  |  |  |  |  |  |  |  |  |  |  |  |  |  |  |  |  |  |  |  |  |
| --- | --- | --- | --- | --- | --- | --- | --- | --- | --- | --- | --- | --- | --- | --- | --- | --- | --- | --- | --- | --- | --- | --- | --- | --- | --- | --- | --- | --- | --- | --- | --- | --- | --- | --- | --- | --- | --- | --- | --- | --- | --- | --- | --- | --- | --- | --- | --- | --- | --- | --- | --- | --- | --- | --- | --- | --- | --- | --- | --- | --- | --- | --- | --- | --- | --- | --- | --- | --- | --- | --- | --- | --- | --- | --- | --- | --- | --- | --- | --- | --- |
|  | H | | | R^2^ | | | CC | | | LL | | | UL | | |  |  |  |  |  |  |  |  |  |  |  |  |  |  |  |  |  |  |  |  |  |  |  |  |  |  |  |  |  |  |  |  |  |  |  |  |  |  |  |  |  |  |  |  |  |  |  |  |  |  |  |  |  |  |  |  |  |  |  |  |  |  |  |  |  |
| LF_S | II | | | 0.48 | | | 4 | | | 1 | | | 8 | | |  |  |  |  |  |  |  |  |  |  |  |  |  |  |  |  |  |  |  |  |  |  |  |  |  |  |  |  |  |  |  |  |  |  |  |  |  |  |  |  |  |  |  |  |  |  |  |  |  |  |  |  |  |  |  |  |  |  |  |  |  |  |  |  |  |
|  |  | | | 40th Percentile | | | | | | | | | | | | 20th Percentile | | | | | | | | | | | | | | | | 80th Percentile | | | | | | | | | | | | | | | | | | | | 90th Percentile | | | | | | | | | | | | | | | | |  |  |  |  |  |  |  |  |  |  |  |  |
| RF_S | IV | | | 0,54 | | | 2 | | | 1 | | | 3 | | |  | | | |  | | | |  | | | |  | | | | |  | | | |  | | | |  | | | |  | | | | | | | |  | | | |  | | |  | | |  | | | | | | |  |  |  |  |  |  |  |  |  |  |  |
|  | III | | |  | | |  | | |  | | |  | | |  | | | |  | | | |  | | | |  | | | | |  | | | |  | | | |  | | | |  | | | | | | | | 0,45 | | | | *4* | | | 0 | | | 8 | | | | | | |  |  |  |  |  |  |  |  |  |  |  |
|  | II | | |  | | |  | | |  | | |  | | |  | | | |  | | | |  | | | |  | | | | | 0,47 | | | | *4* | | | | *1* | | | | *7* | | | | | | | |  | | | |  | | |  | | |  | | | | | | |  |  |  |  |  |  |  |  |  |  |  |
|  | I | | |  | | |  | | |  | | |  | | | 0,73 | | | | *-4* | | | | -6 | | | | -2 | | | | |  | | | |  | | | |  | | | |  | | | | | | | | 0,48 | | | | *1* | | | 0 | | | 2 | | | | | | |  |  |  |  |  |  |  |  |  |  |  |
|  |  | | | 40th Percentile | | | | | | | | | | | | 50th Percentile | | | | | | | | | | | | | | | | 60th Percentile | | | | | | | | | | | | | | | | | | | 70th Percentile | | | | | | | | | | | | | | | | | 80th Percentile | | | | | | 90th Percentile | | | | |  | |
| LH_S | III | | | 0,49 | | | *-20* | | | -37 | | | -3 | | | 0,53 | | | | *5* | | | | *1* | | | | *8* | | | | | 0,52 | | | | 3 | | | | *1* | | | | 6 | | | | | | | | 0,77 | | | | *3* | | | 2 | | | 5 | | | | | | | 0,65 | 4 | 2 | 6 | | 0,48 | 3 | 0 | 5 | |  |
| RH_S |  | | | 50th Percentile | | | | | | | | | | | | 70th Percentile | | | | | | | | | | | | | | | | 80th Percentile | | | | | | | | | | | | | | | | | |  |  |  |  |  |  |  |  |  |  |  |  |  |  |  |  |  |  |  |  |  |  |  |  |  |  |  |  |  |  |  |
|  | III | | |  | | |  | | |  | | |  | | | 0,42 | | | | *2* | | | | 0 | | | | 4 | | | | | | 0,47 | | | | *2* | | | | *0* | | | | *4* | | | | | | | |  |  |  |  |  |  |  |  |  |  |  |  |  |  |  |  |  |  |  |  |  |  |  |  |  |  |  |
|  | II | | | 0,48 | | | *10* | | | 1 | | | 19 | | | 0,66 | | | | *4* | | | | 2 | | | | 6 | | | | | |  | | | |  | | | |  | | | |  | | | | | | | |  |  |  |  |  |  |  |  |  |  |  |  |  |  |  |  |  |  |  |  |  |  |  |  |  |  |  |
| LF_F |  | | | 10th Percentile | | | | | | | | | | | | 40th Percentile | | | | | | | | | | | | | | | | 60th Percentile | | | | | | | | | | | | | | | | | | 90th Percentile | | | | | | | | | | | | | | | | |  |  |  |  |  |  |  |  |  |  |  |  |  |  |
|  | IV | 0,48 | | | *-1* | | | -3 | | | 0 | | | 0,74 | | | *-55* | | | | -79 | | | | -31 | | | |  | | | | | | |  | | | |  | | | |  | | | | | 0,48 | | | | | | | 2 | | | *1* | | | 4 | | | |  |  |  |  |  |  |  |  |  |  |  |  |  |  |  |
|  | III | 43 | | | *-3* | | | -5 | | | 0 | | |  | | |  | | | |  | | | |  | | | | 0,48 | | | | | | | *10* | | | | *2* | | | | *18* | | | | |  | | | | | | |  | | |  | | |  | | | |  |  |  |  |  |  |  |  |  |  |  |  |  |  |  |
|  | II |  | | |  | | |  | | |  | | |  | | |  | | | |  | | | |  | | | | 0,67 | | | | | | | *-11* | | | | *-17* | | | | *-5* | | | | |  | | | | | | |  | | |  | | |  | | | |  |  |  |  |  |  |  |  |  |  |  |  |  |  |  |
| RF_F |  | 10th Percentile | | | | | | | | | | | | 90th Percentile | | | | | | | | | | | | | | | | |  |  |  |  |  |  |  |  |  |  |  |  |  |  |  |  |  |  |  |  |  |  |  |  |  |  |  |  |  |  |  |  |  |  |  |  |  |  |  |  |  |  |  |  |  |  |  |  |  |  |
|  | II | 0,46 | | | *-2* | | | -3 | | | 0 | | |  | | |  | | | |  | | | |  | | | |  |  |  |  |  |  |  |  |  |  |  |  |  |  |  |  |  |  |  |  |  |  |  |  |  |  |  |  |  |  |  |  |  |  |  |  |  |  |  |  |  |  |  |  |  |  |  |  |  |  |  |  |
|  | I | 0,51 | | | *-4* | | | -7 | | | 1 | | | 0,73 | | | *3* | | | | 2 | | | | 4 | | | |  |  |  |  |  |  |  |  |  |  |  |  |  |  |  |  |  |  |  |  |  |  |  |  |  |  |  |  |  |  |  |  |  |  |  |  |  |  |  |  |  |  |  |  |  |  |  |  |  |  |  |  |
| LH_F |  | 50th Percentile | | | | | | | | | | | | 70th Percentile | | | | | | | | | | | | | | | | | 80th Percentile | | | | | | | | | | | | | | | | | 90th Percentile | | | | | | | | | | | | | | | | | |  |  |  |  |  |  |  |  |  |  |  |  |  |  |  |
|  | IV |  | | |  | | |  | | |  | | | 0,53 | | | | | *3* | | | | 1 | | | | 5 | | | | 0,45 | | | | | *2* | | | | 0 | | | | 3 | | | | 0,56 | | | | | | | | *2* | | | *1* | | | *3* | | | |  |  |  |  |  |  |  |  |  |  |  |  |  |  |  |
|  | II | 62 | | | 4 | | | *1* | | | 6 | | |  | | | | |  | | | |  | | | |  | | | |  | | | | |  | | | |  | | | |  | | | |  | | | | | | | |  | | |  | | |  | | | |  |  |  |  |  |  |  |  |  |  |  |  |  |  |  |
| RH_F |  | | 10th Percentile | | | | | | | | | | | | 70th Percentile | | | | | | | | | | | | | | | 80th Percentile | | | | | | | | | | | | | | | | | 90th Percentile | | | | | | | | | | | | | | | | |  | | | | | | | | | | | | | | | | |
|  | IV | | 0,59 | | | *-4* | | | -6 | | | -1 | | | 53 | | | *4* | | | | 1 | | | | 7 | | | | 0,89 | | | | | *5* | | | | *3* | | | | *6* | | | | 0,62 | | | | | | | | 5 | | | *2* | | | 9 | | | |  |  |  |  |  |  |  |  |  |  |  |  |  |  |  |  |
|  | III | | 0,51 | | | *-3* | | | -5 | | | -1 | | |  | | |  | | | |  | | | |  | | | |  | | | | |  | | | |  | | | |  | | | |  | | | | | | | |  | | |  | | |  | | | |  |  |  |  |  |  |  |  |  |  |  |  |  |  |  |  |
|  | II | | 0,43 | | | *-2* | | | -4 | | | 0 | | |  | | |  | | | |  | | | |  | | | |  | | | | |  | | | |  | | | |  | | | |  | | | | | | | |  | | |  | | |  | | | |  |  |  |  |  |  |  |  |  |  |  |  |  |  |  |  |
|  | I | |  | | |  | | |  | | |  | | |  | | |  | | | |  | | | |  | | | | 0,42 | | | | | *3* | | | | *0* | | | | *6* | | | |  | | | | | | | |  | | |  | | |  | | | |  |  |  |  |  |  |  |  |  |  |  |  |  |  |  |  |
